# Supplementary material for: Polyketide synthase gene clusters in the ericoid mycorrhizal fungus Oidiodendron maius: genome-wide prediction, expression profiling, and functional characterization of knockout mutants
Source: Front Plant Sci. 2026 Sep 16;17:1924430. doi: 10.3389/fpls.2026.1924430 (PMC13623903; doi:10.3389/fpls.2026.1924430)
Supplement: Supplementary file 1 [file SupplementaryFile1.zip › Supplementary Materials/Supplementary Folder 1.zip!/Oidma1_AssemblyScaffolds_Repeatmasked/knownclusterblast/cluster4/jgi.p_Oidma1_70428_mibig_hits.html]

| MIBiG Protein | Description | MIBiG Cluster | MiBiG Product | % ID | % Coverage | BLAST Score | E-value |
| --- | --- | --- | --- | --- | --- | --- | --- |
| EAU38785.1 | predicted\_protein | BGC0000161 | Terrein\_biosynthetic\_gene\_cluster | 39 | 103.47107438 | 369 | 7.2e-102 |
| ACB12566.1 | Fum21 | BGC0000063 | Fumonisin\_biosynthetic\_gene\_cluster | 24 | 102.809917355 | 107 | 6.7e-23 |
